# Regulation of the Cell-Intrinsic DNA Damage Response by the Innate Immune Machinery

**DOI:** 10.3390/ijms222312761

**Published:** 2021-11-25

**Authors:** Thomas J. Hayman, Peter M. Glazer

**Affiliations:** 1 Department of Therapeutic Radiology, Yale University School of Medicine, New Haven, CT 06510, USA; thomas.hayman@yale.edu; 2 Department of Genetics, Yale University School of Medicine, New Haven, CT 06510, USA

**Keywords:** DNA damage response, STING, cGAS, DNA repair, radiation, innate immune system

## Abstract

Maintenance of genomic integrity is crucial for cell survival. As such, elegant DNA damage response (DDR) systems have evolved to ensure proper repair of DNA double-strand breaks (DSBs) and other lesions that threaten genomic integrity. Towards this end, most therapeutic studies have focused on understanding of the canonical DNA DSB repair pathways to enhance the efficacy of DNA-damaging therapies. While these approaches have been fruitful, there has been relatively limited success to date and potential for significant normal tissue toxicity. With the advent of novel immunotherapies, there has been interest in understanding the interactions of radiation therapy with the innate and adaptive immune responses, with the ultimate goal of enhancing treatment efficacy. While a substantial body of work has demonstrated control of the immune-mediated (extrinsic) responses to DNA-damaging therapies by several innate immune pathways (e.g., cGAS–STING and RIG-I), emerging work demonstrates an underappreciated role of the innate immune machinery in directly regulating tumor cell-intrinsic/cell-autonomous responses to DNA damage.

## 1. Introduction

On a daily basis, every cell in the human body receives >10^4^ DNA lesions from both exogenous and endogenous insults such as radiation, carcinogens and replication stress [1,2,3]. Unless these DNA lesions are repaired in a timely manner, they may lead to impaired replication, transcription and ultimately genomic instability and threatened cellular viability. As such, maintenance of genomic integrity is of critical importance for organismal survival and cells have thus evolved to contain a sophisticated DNA damage response (DDR) system responsible for both DNA lesion sensing as well as repair of these lesions. DNA double-strand breaks (DSBs) are a particularly lethal version of DNA damage that are primarily repaired by either of two major DSB repair pathways: homologous recombination (HR) or non-homologous end-joining (NHEJ) [4]. NHEJ is an error-prone process that occurs throughout the cell cycle and ligates DNA ends without the presence of a donor template, whereas HR is an error-free pathway that requires an intact sister chromatid to serve as an accurate repair template and occurs primarily in the S and G2 cell cycle phases [4,5].

Both of the canonical DNA DSB repair pathways are crucial for a cell’s ability to maintain genomic integrity [6,7]. Alterations to these pathways leads to accumulation of DNA damage, subsequent mutations and promotion of an undesired phenotype, cell death or tumorigenesis. From a therapeutic perspective, the most commonly used cancer therapies (e.g., radiation therapy and numerous systemic therapies) exert their anti-cancer properties through generation of DNA DSBs [8]. As such, there has been considerable pre-clinical and clinical effort devoted to the study of the canonical DNA DSB repair pathways with the goal of developing potential targets to enhance therapeutic efficacy. Specifically, central effector molecules in the DDR such as ATM (ataxia-telangiectasia mutated), ATR (ataxia-telangiectasia mutated and Rad3 related) and their downstream effectors (e.g., CHK1 and CHK2) have been extensively studied with multiple pharmacologic agents developed to inhibit their activity [2,9,10]. Additionally, there are multiple tumor types that have been discovered to harbor alterations in DNA repair pathways, such as homologous recombination. For instance, in the 1990s, *BRCA1* and *BRCA2* were identified as tumor suppressor genes responsible for a significant proportion of hereditary breast cancers and their inactivation leads to a HR-deficient (HRD) state [11,12,13,14,15]. It has subsequently been well established that tumors exhibiting HRD have sensitivity to platinum agents as well as other DDR-targeted therapies [10,16]. While these efforts have been fruitful, many of these agents have been relatively limited in efficacy and have potentially significant normal tissue toxicity.

With the advent of successful immunotherapies in oncology, there has been significant reinvigoration and increased study of the crosstalk between the immune system and the anti-tumor response to conventional cancer-directed therapies such as radiation therapy and chemotherapy [17,18]. The evidence for crosstalk between the DDR and the innate and adaptive immune systems is complex and evidenced in several physiologic and pathologic states. For instance, in developing B and T cells, the process of V(D)J recombination requires the production and repair of targeted DNA DSBs at the antigen receptor loci [19]. Generation of these DSBs leads to binding and activation of canonical DDR proteins such as Ku70, Ku80, DNA-PKcs, ATM, and the MRN (MRE11, Rad50, NBS1), followed by repair of these DSBs and ultimately the generation of antigen receptor diversity [19,20,21]. Immunoglobulin class switch recombination (CSR) in activated, mature B cells allows the generation of antibodies with different isotypes [22]. Through a process of closely staggered single-strand nicks on alternative strands DNA DSBs are formed [19,23]. The DNA ends of the DSBs in different switch regions are then joined either through classical or alternative NHEJ to facilitate immunoglobulin class switching [6,19,24].

In the context of infection, it has also been long recognized that foreign DNA elicits a host immune response. One of the earliest reports was in 1963, when Isaacs et al. noted that in mouse cells infected with chick nucleic acid, there was production of interferons (IFNs) [25]. Furthermore, it has been documented that upon infection with and replication of multiple virus types there is induction of DNA damage in the host cell [26]. Adenovirus (Ad)12 infection of human embryonic kidney cells induces chromosomal aberrations and chromosomal breaks [27,28,29]. This has also been demonstrated for other DNA viruses such as HSV, hepatitis B virus (HBV), and simian virus 40 (SV4) [26]. To successfully undergo viral replication, retroviruses, a unique subtype of virus that includes HIV1, must transcribe their RNA genome into DNA followed by integration into the host genome using an integrase [30]. During integration, DNA DSBs are formed and efficient repair of these lesions is critical for viral replication and host cell survival [31]. This repair is dependent upon NHEJ and other DNA repair pathways [31,32,33,34,35]. Similarly, Influenza A virus has also been shown to induce direct DNA damage upon infection and replication and this DNA damage must be repaired to ensure host cell survival and subsequent viral replication [36]. Additionally, during replication, multiple viruses can modulate the function of DDR proteins. For instance, adenoviruses cause E3-mediated degradation of DDR proteins [37]. SV40 also initiates the degradation of MRE11, a protein critical for the DNA DSB detection [38]. These results highlight the multifaceted interaction of the host cell-intrinsic DDR and viral infection.

The innate immune system is responsible for the detection of foreign DNA and RNA followed by activation of an immune response typically through production of cytokines and other immune stimulated genes (ISGs). This process typically involves pattern recognition receptors (PRRs) that detect microbial products (PAMPs; pathogen associated molecular patterns) or products from dying cells (DAMPs; damage-associated molecular patterns) [39,40]. PRRs include Toll-like receptors (TLRs), retinoic acid-inducible gene I (RIG-I)-like receptors (RLRs) and nucleotide-binding oligomerization domain (NOD)-like receptors [41,42]. The RLRs family of PRRs include RIG-I and MDA5 and function to detect viral replication in the cytosol and/or nucleus [43,44]. There is considerable crosstalk between these pathways and their signaling pathways are reviewed in detail elsewhere [45]. We will specifically focus on the discussion of a few of these pathways given their demonstrated relevance in the anti-tumor response and crosstalk with the DDR.

RIG-I is a key cytosolic viral RNA sensor that is responsible for the detection of multiple viral RNAs. Upon recognition and binding to RNA, RIG-I recruits the mitochondrial anti-viral-signaling protein (MAVs) via its caspase activation recruitment domain (CARD) [46]. This induces the formation of filamentous oligomers of MAVS CARD on the mitochondrial surface. This activation of MAVS leads to the recruitment of downstream signaling molecules including IRF3/7 and NF-kB signaling leading to a robust anti-viral interferon response through production of IFN-beta and other inflammatory cytokines [42,47].

The cyclic guanosine monophosphate (GMP)-adenosine monophosphate synthase (cGAS)-stimulator of interferon genes (STING) signaling pathway is a critical bridge that functions to detect viral pathogens and mount and anti-viral immune response [48,49,50]. It was first defined as a cytosolic PRR that detects pathogen-derived DNA that is released into the cytosol of cells during an infection [50,51]. cGAS binds to this pathogen DNA, followed by a conformation change in cGAS that allows cGAS to catalyze ATP and GTP into the 2′3′-cyclic GMP-AMP (cGAMP), a cyclic dinucleotide, that contains both a 2′-5′ and 3′-5′ phosphodiester linkage [52,53,54,55]. cGAMP then acts as a second messenger that binds to and stimulates the endoplasmic reticulum (ER)-resident protein STING [56,57,58]. Upon binding of cGAMP, STING oligomerizes and undergoes translocation from the ER to the Golgi apparatus. Upon its palmitoylation in the Golgi, STING then recruits TBK1 (TANK-binding kinase 1) and TBK1 phosphorylates STING allowing for recruitment of IRF3 (interferon regulatory factor 3) [58,59,60]. Activated IRF3 then translocates to the nucleus where it acts as a transcription factor to enhance transcription of type 1 IFNs and ISGs [48,58]. Beyond TBK1/IRF3, STING activation also leads to activation of IKK and NF-kB to drive transcription of NF-kB-dependent genes [58,61]. Following activation, STING is then degraded by trafficking to the endo-lysosomes to prevent sustained activation [62].

As opposed to non-malignant cells, tumor cells have a propensity to contain cytosolic DNA where it is in turn recognized by cGAS given its affinity for dsDNA in a sequence-independent manner [55,63]. Generation of cytosolic DNA occurs from multiple sources within the cells including the nucleus (e.g., genomic DNA) and the mitochondria. Due to frequent oxidative stress and mitochondrial dysfunction, there is release of mtDNA into the cytosol where cGAS is able to recognize this and activate STING signaling. This has been demonstrated in KRAS-LKB1 mutant NSCLC cells and in non-malignant states as well. In addition, genomic and chromosomal instability (CIN) are hallmarks of cancer cells [64]. Multiple studies have demonstrated that these properties of tumor cells lead to production of micronuclei, structures that are prone to envelope rupture, thus exposing their nuclear/genomic DNA contents to cGAS [18,65,66,67]. This in turn leads to activation of the cGAS–STING signaling axis and production of IFNs and other ISGs leading to inflammation and a host of other cellular phenotypes.

Beyond tumor cell-intrinsic genomic instability and CIN, exogenous DNA-damaging stimuli (e.g., radiation, cisplatin, etoposide and PARP inhibitors) and are able to increase the generation of cytosolic DNA and RNA, thus engaging either the cGAS–STING or RIG-I signaling pathways [68,69,70,71,72,73,74,75]. These responses have been shown to be important components of that anti-tumor immune responses to these therapies. Specifically, Harding et al. show that after radiotherapy, progression through the cell cycle leads to the generation of micronuclei, followed by cGAS recognition and generation of inflammatory responses [65]. These inflammatory responses were dependent upon mitotic progression and cGAS–STING signaling and drove STING-dependent abscopal responses in the context of radiation and immunotherapy treatment. In addition, Deng et al. demonstrate that in response to high-dose, single-fraction radiation, stromal STING was essential for anti-tumor responses to radiation in pre-clinical MC38 tumor xenografts [68]. This response was shown to be reliant on production of Type I IFNs in dendritic cells (DC) in response to irradiated tumor cells in a cGAS–STING-dependent manner. This STING-dependent DC response leads to the generation of a robust CD8+ T-cell-dependent adaptive immune response that was necessary for the anti-tumor effects of radiotherapy in this model. Similar results have been documented for cisplatin and other DNA-damaging chemotherapies, where DNA damage leads to STING-dependent signaling and activation of a CD8+ T-cell anti-tumor immune response [70,73].

Interestingly, there has been suggestion of an interplay between stromal (or microenvironmental) and tumor cGAS–STING signaling with regard to regulating the immune-based anti-tumor response to DNA-damaging therapies. In the context of PARP inhibitors, it was shown that in TNBC (triple-negative breast cancer) pre-clinical models, olaparib treatment induced CD8+ T-cell infiltration and activation that was necessary for its anti-tumor activity [73]. In contrast to prior work demonstrating the importance of stromal cGAS–STING signaling in regulating the anti-tumor response to DNA-damaging therapies, this group demonstrated that the anti-tumor effects of olaparib required tumor cell cGAS–STING activation. Specifically, they showed that olaparib treatment induced cGAS–STING activation in breast cancer cells led to a paracrine activation of DCs and thus induction of a robust anti-tumor CD8+ adaptive immune response. Importantly genetic depletion of tumor cGAS–STING abolished the CD8+ adaptive immune response and anti-tumor activity of olaparib. In the context of radiotherapy, similar stromal and tumor cGAS–STING signaling crosstalk is beginning to come to light. Carozza et al. show that tumor cell production of cGAMP is required for the anti-tumor effects of radiotherapy in their in vivo models [76]. They demonstrate radiation-induced production of cGAMP (via tumor cGAS) leads to its secretion into the extracellular space. cGAMP is then sensed by host/stromal STING where it stimulates a CD8+ T-cell response that is responsible for the anti-tumor efficacy of ionizing radiotherapy. Furthermore, in this study the authors develop a small-molecule inhibitor of ENPP1 (ectonucleotide pyrophosphatase phosphodiesterase 1), the endogenous cGAMP hydrolase, that when delivered increases extracellular cGAMP concentrations and enhances the efficacy of radiotherapy in pre-clinical mouse models of breast cancer. The results of these studies begin to highlight the underappreciated role of tumor intrinsic STING with stromal cGAS–STING crosstalk in regulating the response to DNA-damaging therapies.

The significant body of work described above showing that stromal cGAS–STING activation in response to DNA damage leads to anti-tumor immune responses has led to enthusiasm in terms of pharmacologically activating this pathway to enhance therapeutic efficacy and tumor control both pre-clinically and clinically. Initial clinical and pre-clinical work has focused upon using STING agonists to enhance responses to other immunotherapies (e.g., PD1/PD-L1 axis agents) or as a monotherapy [77,78,79]. However, testing of STING pathway agonists as monotherapies (or in combination with PD1 axis agents) has shown disappointing results. Interim data using the intratumoral STING agonist MK-145 demonstrated no antitumor responses as a monotherapy [80]. However, pre-clinical would suggest that perhaps an alternative approach entails combinations with DNA-damaging agents. Deng et al. demonstrate that activation of stromal STING through use of the endogenous STING ligand, cGAMP, enhances the efficacy of radiotherapy in MC38 colon cancer xenografts [68]. Another group has shown that intratumoral injection of a proprietary STING agonist, RR-CDG, enhances the efficacy of radiotherapy both locally and in an abscopal manner in pre-clinical pancreas cancer models. Again, this response was shown to be dependent upon stromal STING and subsequent CD8+ T-cell response [81]. Similar results were obtained with inhalable nanoparticle STING agonists in combination with radiotherapy in lung cancer metastases [79]. This has led to the advancement of intratumoral STING agonists in combination with DNA-damaging therapies in the clinical trial setting, with an emphasis on enhancing immune-based or abscopal type radiation responses.

In addition to the activation of cGAS–STING and other PRRs by DNA damage, there are several lines of evidence that highlight a reciprocal relationship between the DDR and innate immune response (i.e., changes in DDR signaling leading to modulation of the immune response). For example, Zhang et al. showed that in pancreatic cancer models, inhibition of ATM leads to increased Type I interferon signaling in a cGAS–STING-independent fashion, resulting in enhanced PD-L1 expression, increased CD8+ T-cell infiltration and ultimately in improved responses to immune checkpoint therapy [82]. Additionally defects in DDR proteins (Rad51, BRCA2, and MLH1) and their associated signaling leads to activation of an immune response through both STING-dependent and STING-independent mechanisms [83,84,85,86]. These data highlight the multifaceted interaction between the immune response and the cellular DDR.

As described above, there is a substantial body of literature detailing the role of cGAS/STING and other TLRs in the regulating the anti-tumor response to DNA-damaging therapies primarily through control of the adaptive immune response ultimately leading to CD8+ T-cell-mediated tumor cell death [68,87]. This area is clearly ripe for continued investigation, both clinically and pre-clinically and may lead to successful clinical strategies to enhance care for patients with various malignancies. However, it has also recently come to light that beyond their canonical role in regulating immune responses, multiple components of the innate immune machinery may be directly involved in regulating tumor cell-intrinsic/cell-autonomous responses to DNA damage and other forms of genotoxic stress. This newfound understanding may lead to a novel understanding of interactions between the immune machinery and tumor cells with the potential to highlight novel therapeutic approaches. As such, the remainder of this review will therefore focus on describing the current understanding of the innate immune machinery and its recently identified role in regulating the tumor cell-intrinsic responses to DNA-damaging therapies and other genotoxic stresses.

## 2. RLRs and the Cell-Intrinsic DNA Damage Response

As previously mentioned, the RNA-sensing pathway involves several RLRs including RIG-I and MDA5 [46]. In addition to RIG-I and MDA5, the DEXH box RNA helicase LGP2 (*DHX58*) is a third cytoplasmic RLR that functions in a multifaceted way to either suppress RIG-I-induced IFN signaling or enhance MDA5-specific anti-viral responses [46]. In contrast to RIG-I and MDA5, LGP2 lacks a CARD domain, and it has been shown to exert its complex regulatory effects through a protein-protein interaction with PACT, an interferon-inducible RNA-binding protein [88]. In the context of the cell-intrinsic radiation response Widau et al. utilized a siRNA screen targeting 89 ISGs in 14 different cancer cell lines and identified LGP2 as a regulator of cell death following exposure to radiation [89]. Specifically, when LGP2 expression was reduced using RNAi they showed increased sensitivity to ionizing radiation as measured by the clonogenic survival assay and conversely with overexpression of LGP2 leading to radiation resistance. They then show that radiation treatment induced LGP2 expression and IFN production. These effects were then connected to control of radiation sensitivity with a series of experiments demonstrating partial control of the radiation response by LGP2 through control of cytotoxic IFN-beta production. Specifically, partial restoration of viability in cell lines with LGP2 knockdown by the addition of an IFN neutralizing antibody with radiotherapy was demonstrated. These were the first results beginning to suggest that control of the RNA sensing pathway may control the tumor cell-intrinsic DNA damage response partially through modulation of cytokine secretion.

Given the role of LGP2 in functioning as a suppressor of the RNA-sensing pathway, Ranoa et al. then sought to define the role of individual members of the RNA-sensing pathway (e.g., RIG-I, MDA5 and MAVS) on the radiation response using [75]. RNAi-mediated knockdown of MAVS increased cell-intrinsic resistance to radiotherapy as measured by the clonogenic survival assay and measures of apoptosis (i.e., caspase activity). Similar results were obtained in immunodeficient mouse xenograft studies, highlighting an immune-independent control of the radiation response. They also show that MAVS knockdown reduced IFN-beta production in response to radiation. Both RIG-I and MDA5 function as RLRs and exert their activity through MAVS. RIG-I knockdown increased resistance to radiation and blunted the radiation-induced secretion of IFNs, whereas MDA5 knockdown had no impact on IFN production or sensitivity to radiation. They then further demonstrate an interaction between RIG-I and endogenous dsRNA in the context of radiation. Taken together, these data suggest that RIG-I/MAVS were responsible for RNA sensing in the context of radiation therapy. While the exact mechanism by which RIG-I and MAVS impact the cell-intrinsic/cell-autonomous radiation response was not defined in this study, it does highlight the role of this pathway in this regard and suggests that enhancement of this pathway may serve as a potential therapeutic strategy in combination with DNA-damaging agents.

A recent publication further explored the role of RIG-I in regulating the cell-intrinsic response to DNA damage [90]. They show that RIG-I is recruited to sites of DNA DSBs and functions to suppress NHEJ-mediated repair of these DSBs, leading to increased DSBs after radiation treatment. In contrast, there were no effects on HR-mediated DNA repair by RIG-I. Furthermore, manipulation of MDA5 and MAVs had no effects on NHEJ. This effect was mediated through RIG-I recruitment to sites of DNA DSBs through interaction with XRCC4, a protein that functions together with Ligase IV (LIG4) to perform DNA ligation during NEHJ. They then proceed to demonstrate that RIG-I functions to suppress NHEJ by inhibiting the formation of the XRCC4/LIG4/XLF complex. Intriguingly, RIG-I agonists suppressed NHEJ and further decreased the chromatin association of LIG4 and XLF in a RIG-I-dependent manner. From a functional perspective, knockdown of RIG-I imparted tumor cell-intrinsic resistance to radiation in the clonogenic survival assay as well as in xenografts generated from WT and RIG-I knockdown tumor cells implanted in immunodeficient mice. While it may initially be unclear why a component of the RNA-sensing innate immune machinery would be involved directly in DNA repair, it is important to remember that certain viruses such as retroviruses must integrate into the host genome and that NHEJ plays a critical role in the regulation of retroviral integration into the host genome [31,91]. As such, the authors then show that RIG-I overexpression suppresses retrovirus genomic integration in an XRCC4-dependent manner, suggesting that this phenomenon is dependent upon RIG-I’s control of NHEJ.

In summary, these studies suggest that beyond its canonical role in promoting a Type I IFN response to inhibit RNA virus replication, RIG-I cooperates with XRCC4 to suppress viral integration into the host-genome in a manner that is dependent upon its suppression of NHEJ-based DNA repair. This has potential significant implications to tumor biology and suggests that RIG-I agonist-based therapies may be a potential avenue to improve responses to DNA-damaging therapies (i.e., ionizing radiation or other systemic therapies) and deserves further study both pre-clinically and clinically.

## 3. cGAS–STING and Cellular Senescence

Senescence is defined as a state of permanent cell-cycle/growth arrest in cells that occurs after various forms of stress including DNA damage, aging, or oncogene-induced stress [92]. Several recent studies have linked cGAS–STING signaling to control of cellular senescence as well as the senescence associate secretory phenotype (SASP) that is responsible for a pro-inflammatory phenotype with both pro and anti-tumor effects [93,94,95]. In the context of irradiation and other DNA-damaging therapies, induction of senescence is considered a form of clonogenic cell death as cells are no longer able to reproduce and give rise to daughter progeny and is therefore of interest as a therapeutically relevant strategy to enhance the effects of genotoxic cancer therapies [96].

Gluck et al. reported that innate immune sensing through cGAS–STING signaling was responsible for senescence induction in a paracrine fashion [54]. This observation was first noted through decreased age-induced senescence in normal cells in tissue culture with cGAS and STING knockout (KO) and was also observed in the context of oxidative stress (culture in high oxygen conditions). Regulation of senescence was then defined to occur through paracrine secretion of SASP components (e.g., IL-6 and Cxcl2) where conditioned media from senescent cells could induce senescence in untreated WT or cGAS KO cells. With regard to the regulation of genotoxic stress-induced senescence, cGAS and STING depletion only partially influenced radiation-induced senescence, likely suggesting that radiation induces senescence in these cells only partially through a paracrine-dependent mechanism. These data suggest that cGAS/STING signaling may function to enhance or propagate senescence after stress through a paracrine fashion.

A separate study by Yang et al. was also published describing the role of cGAS in regulating cellular senescence almost simultaneously [94]. They also described the role of cGAS in promoting senescence during the spontaneous immortalization of murine embryonic fibroblasts. Interestingly, they showed that deletion of STING had no effects on immortalization-induced senescence, suggest a STING-independent function in their model for cGAS-mediated senescence. They further go on to demonstrate that deletion of cGAS in both normal cell lines and murine melanoma cell lines that cGAS is essential for DNA damage-induced senescence (etoposide and radiation). They then go on to show that in a cohort of NSCLC patients low cGAS expression correlates with reduced overall survival potentially suggesting a role for loss of a senescent phenotype and tumorigenesis, malignant progression, or resistance to therapy-induced senescence.

A third study by Dou et al. has also examined the role of cGAS–STING in regulating cellular senescence and SASP [93]. They show that cGAS–STING signaling, through interactions with cytoplasmic chromatin fragments, also promotes SASP after DNA damage (or other stimuli) through NF-kB, but not IRF3 suggesting that there may be unique downstream signaling specific to SASP. Depletion of STING was then noted to decrease tissue inflammation through a diminished SASP and that tumor cells have an active pro-inflammatory phenotype dependent upon this cGAS–STING driven SASP.

Taken together, these studies would suggest that cGAS–STING-dependent control of senescence in response to genotoxic stress may be an actionable finding whereby strategies aimed at enhancing this response in the short term could induce DNA damage-induced senescence and the pro-inflammatory SASP, theoretically improving the cell-intrinsic response as well as the anti-tumor immune response by activating an adaptive immune response. However, it is worth noting that there have been several lines of evidence that suggest that chronic activation of SASP or cGAS–STING signaling leads to a phenotype of immunosuppression, increased tumor progression, and metastasis [55,63,93,97]. Therefore, it stands to reason that should this develop into a therapeutic strategy care will need to be taken to ensure that pharmacologic induction of cGAS–STING signaling may need to be short term in nature and therefore merits further pre-clinical study.

## 4. STING-Independent Nuclear Functions of cGAS

Several studies began to note localization of cGAS to the nucleus and micronuclei cGAS hinting at non-canonical/STING-independent functions of cGAS that exist outside of its role as a cytoplasmic DNA sensor [98,99,100,101,102,103,104]. Liu et al. demonstrated that upon exposure of cell lines to genotoxic stressors, there was robust nuclear translocation of cGAS even in the context of an intact nuclear membrane [100]. This translocation was independent of cGAS enzymatic and DNA-binding activity, suggesting an uncoupling from its function as a cytosolic DNA sensor. Given its translocation to the nucleus upon DNA damage the role of cGAS in regulating, DNA repair was then investigated and they showed specifically using chromatin immunoprecipitation (CHIP) that cGAS localized to sites of DNA DSBs through a direct interaction with gamma-H2AX. They then demonstrated that nuclear cGAS functions to restrain HR, but has no effects on NHEJ-based DNA repair and that these effects were independent of IFN-beta induction, again suggesting a non-canonical role of cGAS in regulating HR-based DNA repair. The inhibitory effects of cGAS on DNA repair were secondary to an indirect interaction between PARP1 and cGAS. Counterintuitively, cGAS knockdown with RNAi increased sensitivity to DNA-damaging therapies, whereas cGAS overexpression decreased sensitivity to those same therapies. This suggests that changes in survival after cGAS loss may be uncoupled from changes in DNA repair, as cGAS silencing was shown to enhance HR and this change alone would be expected to cause resistance to DNA-damaging therapies rather than sensitivity.

A subsequent study further examined the role of cGAS in regulating DNA repair [101]. They found constitutive presence of cGAS in both the cytosol and nucleus with some cell cycle variation. In contrast to Liu et al., they found that the DNA binding of cGAS was required for nuclear localization at baseline. In their model systems they found that expression of cGAS increased DNA damage-induced micronuclei formation (a marker of genomic instability) and increased cell death as well as bone marrow depletion consistent with cGAS-mediated suppression of DNA repair. This phenomenon was reported to be independent of STING expression. Similar to Liu et al. the effects of cGAS on DNA repair were shown to be primarily through HR rather than NHEJ and that its effects on HR were dependent upon DNA binding and oligomerization but independent of its enzymatic function. They then further explored the mechanism by which cGAS interferes with HR and showed that cGAS acted by impeding the critical HR steps of RAD51-mediated strand invasion and subsequent D-loop (displacement loop) formation through compaction of dsDNA into a higher-ordered state.

These studies share several commonalities including localization of cGAS to the nucleus leading to inhibition of HR-based DNA repair, causing increased genomic instability with a requirement for nuclear localization signals. However, the exact mechanisms by which cGAS interacts with HR appears to be multifaceted and divergent between these studies. Additionally, using different functional readouts, there were divergent effects of cGAS loss on cell death that would not be predicted based solely on its role in regulating DNA repair (as increased cell death following exposure to DNA damage would be expected with cGAS expression due to suppression of HR).

Given these discrepancies, a more recent publication sought to better understand the role of nuclear cGAS in regulating genomic stability and response to genomic stress. Using both untransformed and cancer models they show that loss of cGAS (generated using CRISPR-Cas9 rather than RNAi) leads to increased cellular proliferation in a manner that was dependent upon both nuclear localization and DNA binding and was independent of STING [105]. These changes in cellular proliferation were shown to be related to increased DNA replication in cGAS-deficient cells through the acceleration of replication fork progression. Furthermore, cGAS-deficient cells restart stalled replication forks prematurely and have decreased stability of stalled replication forks suggesting that cGAS functions to impart genomic stability through maintenance of replication fidelity. These replication defects in cGAS-deficient cells led to activation of a replication stress phenotype with an increase in ATR signaling. They then show that this hyperproliferative/hyper-replicative state with cGAS loss increases sensitivity to genotoxic stress (radiation, cisplatin and hydrogen peroxide). The mechanism by which cGAS slows replication was by interacting with chromatin/DNA and acting as a replication “roadblock”.

In summary, these results suggest that outside of its canonical role as a cytosolic DNA sensor, cGAS localizes to the nucleus and functions to regulate genomic stability through multiple avenues. Surprisingly cGAS seems to act in opposing fashions when it comes to regulating genomic stability through suppression of HR-mediated DNA repair (decreased genomic stability) and simultaneously acting to decelerate replication forks and hence increase genomic stability. It remains unclear at this time why these opposing actions may have evolved evolutionarily, but generally these data seem to suggest that overall cGAS expression, both through canonical and non-canonical functions, seems generally to act to promote genomic stability and decrease tumorigenesis. This idea is also generally supported by multiple lines of evidence that show that many tumors have evolved to suppress cGAS expression as a means to escape immune surveillance and evade cGAS-induced cell death.

## 5. STING and Autophagy

Regulation of autophagy has recently been shown to be a primordial function of the cGAS–STING signaling pathway, evolutionarily predating its role in the regulation of interferon signaling [106]. Autophagy is a regulated program by which cytoplasmic substrates including, damaged organelles and various proteins are degraded through the formation of autophagosomes and subsequent fusion of the autophagosome with the lysosome [107,108]. Autophagy occurs basally and can be upregulated during periods of physiologic or extracellular stress where generally in normal cells it functions to maintain normal cellular homeostasis by removing misfolded proteins and damaged organelles. It is a complicated process that has been shown to have dual roles both as a pro-survival or pro-death pathway with regard to tumorigenesis and cancer progression [107,108]. In the anti-viral/cytoplasmic DNA response, Gui et al. have shown that cGAS–STING induces autophagy in a manner that is uncoupled from its interferon-inducing functions [106]. Specifically, they demonstrate that in the context of viral infection or cytosolic DNA presence, cGAS produced cGAMP, triggers translocation of STING from the ER (endoplasmic reticulum) to the ERGIC (endoplasmic reticulum–Golgi intermediate complex) and Golgi that is dependent upon ARF family members. While in the ERGIC, cGAMP-bound STING serves to aid in LC3 recruitment and lipidation, which then targets DNA and pathogens to autophagosomes and ultimate lysosome degradation. This function of STING was independent of TBK1 signaling or the *C*-terminal domain of STING both of which are required for its interferon-stimulatory function. Finally, they show that the anti-viral function of STING in part depends upon its role in promoting cGAMP-induced autophagy.

As mentioned above, autophagy has dual roles (e.g., both pro-survival and pro-death) in the context of responses to cellular stress that may be context specific. Nassour and colleagues defined the role of autophagic cell death in regulating chromosomal instability during replicative crisis [109]. Replicative crisis and p53-dependent senescence are two of the major processes by which tumorigenesis is prevented. Given frequent p53 inactivation and cell cycle checkpoint inactivation, cells continue to proliferate, followed by telomere shortening, telomeric damage and ultimately initiation of replicative crisis [110]. During this process, telomeres fuse resulting in cell death [111]. They show that cells undergoing replicative crisis undergo autophagy and that inhibition of autophagy promotes bypass of crisis-associated death. This occurred in a manner dependent upon induction of telomeric DNA damage. Furthermore, replicative crisis-induced autophagy was dependent upon cGAS–STING signaling with depletion of either cGAS or STING allowing cells to proliferate past crisis and emerge as populations of cells with evidence of chromosomal instability as evidenced by accumulation of chromosomal aberrations.

These results raise several intriguing possibilities. First, autophagy has been implicated in the response to DNA damage and it raises the possibility that cGAS–STING may contribute to DNA damage-induced autophagy outside of just replicative crisis. If this were indeed the case, then perhaps potentiation of this response with a STING agonist may be beneficial in combination with DNA-damaging therapies. Finally, these data again suggest that outside of their roles as central regulators of the immune response, cGAS and STING function as “tumor suppressors” in that they serve to prevent tumorigenesis and chromosomal instability through multiple mechanisms including promotion of autophagy in this context.

## 6. STING and Regulation of the Cell-Intrinsic Response to DNA Damage

As highlighted above, the cGAS–STING signaling pathway has been shown to play an important role in the immune-mediated (extrinsic) response to radiation therapy and other DNA-damaging agents. However, its role in regulating the cell-intrinsic response to genotoxic stress has only recently begun to be appreciated. A recent report by Dunphy et al. demonstrated the non-canonical activation of STING in response to etoposide-induced nuclear DNA damage involving the DNA-binding protein IFI16 and the DNA repair proteins ATM and PARP1 [112]. Specifically, they showed that etoposide-induced DNA damage induces an acute, cell-intrinsic innate immune response that is dependent STING but not cGAS. Damaged DNA is recognized by functional PARP1 and ATM where ATM in turn phosphorylates/activates TRAF6 and p53. In turn, there is interaction between IFI6 and p53 followed by IFI6-dependent delivery of p53 to STING. This ultimately leads to formation of a STING-p53-IFI6-TRAF6 complex that functions to activate NF-kB rather than TBK1-IRF3 signaling, and induction of a robust innate immune response with production of IFNs and other ISGs. These results suggest that outside of STING’s canonical role in the sensing of cytoplasmic DNA it also functions to coordinate a robust transcriptional response to nuclear DNA damage. What remains to be determined is how this response is coordinated with the cytosolic DNA response, whether this is generalizable to other forms of DNA-damaging therapy beyond etoposide, and the functional consequence of this signaling cascade with regard to generation of a functional immune response in vivo. Additionally, given the dependence upon functional p53 for this non-canonical STING response, it may be less relevant in a number of tumors given the propensity for p53 mutation/inactivation that is present in multiple tumor types.

In addition to STING regulating an immune response to DNA damage, recent work has also begun to define the role of STING in regulating the cell-intrinsic response to radiotherapy and other forms of genotoxic stress. We recently published the results of an unbiased whole-genome CRISPR-Cas9 screen aimed at defining novel determinants of the cell-intrinsic DNA damage response [113]. Intriguingly, STING was the top hit from the screen with loss of STING imparting cell-intrinsic resistance to radiation. We showed that CRISPR-mediated depletion of STING imparted resistance to radiation and cisplatin treatment using the colony forming assay. In immunodeficient mice, tumors generated from STING depleted tumor cells were significantly more resistant to fractionated radiation, again highlighting STING-dependent regulation of the tumor intrinsic response to radiation. We also showed that STING regulated a transcriptional program that controls the generation of reactive oxygen species (ROS), and that STING loss altered cellular ROS hemostasis, leading to reduced DNA damage and ultimately therapeutic resistance. Using a next-generation intravenous STING agonist, we demonstrate that pharmacologic activation of STING enhances the effects of ionizing radiation in vivo providing pre-clinical rationale for further evaluation of STING agonists in combination with DNA-damaging therapies. Lastly, in agreement with our data showing STING loss is associated with resistance to DNA damage, analysis of a cohort of patients with head and neck squamous cell carcinoma shows that low tumor STING expression is associated with worse oncologic outcomes. These results suggest that STING controls cell-intrinsic responses to ROS-dependent DNA-damaging therapies (e.g., radiation and cisplatin) in a manner that is distinct from its canonical function as a regulator of the innate immune (extrinsic) response to DNA damage. Furthermore, these results also suggest that tumor STING expression may be a relevant biomarker. Specifically, in tumors with low STING expression, consideration of the use of a non-ROS-dependent therapy such as cetuximab may be warranted as their anti-tumor response was shown to be independent of STING expression.

**Figure 1:**
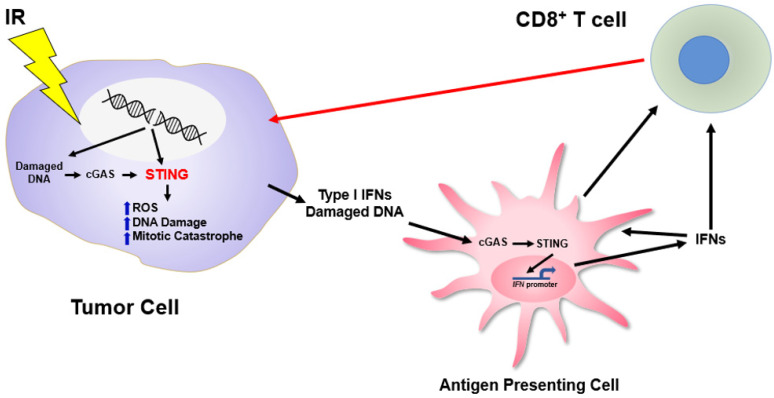
STING-Dependent Regulation of the DNA Damage Response. Ionizing radiation (IR) induces DNA DSBs that subsequently induce cell death in a STING-dependent manner through tumor intrinsic control of ROS, DNA damage and mitotic catastrophe as well as through immune-mediated activation of the CD8 T cells.

Additionally, Ranoa and colleagues elucidated the role of STING controlling cell-cycle progression in a cGAS-independent manner in several pre-clinical models using RNAi-based approaches [114]. They demonstrate that depletion of STING expression using RNAi imparts increased proliferation in vitro and in vivo and that this response in their models was driven by transcriptional changes in genes involved in cell cycle regulation, including CDKN1. Consistent with the above-described results they also show that loss of STING expression imparts resistance to radiation with loss of STING expression being associated with an increase in chromosomal instability as evidenced by increased aneuploidy. Their results describe a dependence upon both p53 and NF-kB for cell-cycle regulation by STING and perhaps this is explained by the aforementioned study describing a p53-dependent NF-kB response to DNA damage that is independent of cGAS. Furthermore, these results may be context specific given the frequent alterations in p53 seen in human tumors and conflicting publications suggesting that STING loss leads to sensitivity to melphalan treatment. Nonetheless, these collective data suggest overall that tumor STING regulates the cell-intrinsic response to DNA damage through mechanisms independent of its canonical cGAS–STING cytosolic DNA-sensing pathway and that activation of this pathway may increase the efficacy of DNA-damaging therapies (Figure 1).

## 7. ISG15 and DNA Replication

Emerging evidence has also implicated additional components of the innate immune response outside of cGAS, STING and RIG-I. One specific example is the protein ISG15. Expression of ISG15 is robustly stimulated by IFNs, viral infection, DNA damage, and cGAS–STING signaling [115,116,117,118]. It is involved in ISGylation, a process similar to ubiquitination, where ISG15 is conjugated to various substrates by the E1 enzyme UBE1L, an E2 ISG15 enzyme UBCH8 and the E3 ligase HERC5 [118]. ISGylation has been shown to affect over 300 proteins with involvement in the anti-viral response and ROS response to viruses [115,116,117,118]. More recently, its role in the DNA damage response has become apparent. Several publications highlight control of ISG15 expression in an IFN-independent manner through activation of p53 upon exposure to irradiation and other forms of DNA-damaging agents as well as telomere shortening [117,119,120]. In the context of the DNA damage response Park et al. examined the role of ISGylation in regulating PCNA [121]. PCNA is known to regulate translesion DNA synthesis in the context of UV-induced damage. They show that ISGylation of PCNA causes termination of the error-prone translesion DNA synthesis and resumption of normal DNA replication, thus preventing the accumulation of excess DNA mutations.

ISG15 was also recently discovered to regulate DNA replication fork progression. This work by Raso and colleagues uses multiple genetic models to manipulate ISG15 expression and demonstrates accelerated replication fork progression in cells expressing higher levels of ISG15 [122]. Interestingly, this effect seemed to be mostly independent of ISG15 conjugation (ISGylation), but rather, was dependent upon interaction of free ISG15 with the DNA helicase RECQ1. In tumor cells with high levels of ISG15 expression and subsequent unrestrained replication fork progression, sensitivity to multiple forms of DNA-damaging agents (camptothecin, cisplatin, and Olaparib) was increased and was associated with a lack of replication fork slowdown after exposure to these agents. This was also associated with an increase in DNA DSBs and chromosomal aberrations and was apparent not only with genetic manipulation of ISG15 expression, but also with IFN-induced ISG15 expression. These results again highlight an emerging area of biology demonstrating control of the cell-intrinsic DNA damage response by canonical components of the innate immune machinery. Additionally, given the role of cGAS–STING in regulating the expression of ISG15 at baseline and in response to DNA damage, this may be another mechanism by which the cGAS–STING signaling pathway regulates tumor intrinsic responses to genotoxic stress and is consistent with prior results showing decreased sensitivity to DNA-damaging therapies with STING loss, given the also demonstrated downregulation of ISG15 in this context. It is also reasonable to conclude that strategies aimed at increasing ISG15 expression may provide a therapeutic vulnerability in context with other genotoxic stress.

## 8. Conclusions

**Figure 2:**
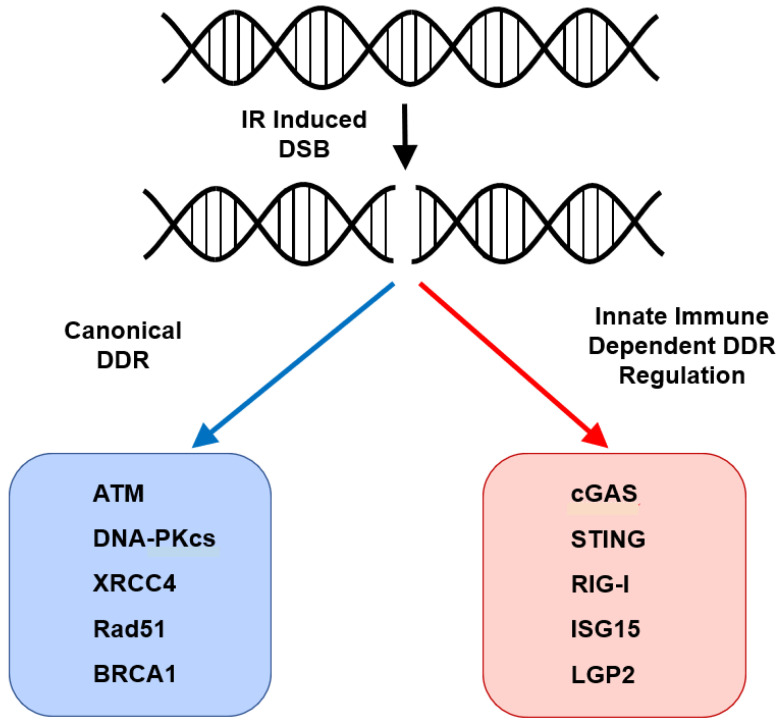
Canonical and non-canonical (innate immune machinery) based regulation of the cell-intrinsic DNA damage response (DDR).

The innate immune system is a complex and diverse system that is poised to direct the immune response to exogenous pathogens by activation of various PRRs and ultimately the adaptive immune response through stimulation of cytokine production. It has a well-established role with regard to its regulation of the cytokine response, activation of T cells and subsequent clearance of various infectious pathogens. As such, its role in regulating the response to cancer-directed therapies, and in particular DNA-damaging therapies, has been most clearly elucidated with respect to regulation of an immune-based response. This has been a large step forward in understanding the response to DNA-damaging therapies and has the potential to inform novel immunotherapy-based regimens for the treatment of malignancies. However, it has become increasingly clear that in addition to regulation of the immune response to DNA-damaging therapies, multiple pathways and their respective components are involved in the regulation of the cell intrinsic/non-immune-mediated regulation of the DNA damage response (Figure 2). This newfound understanding will lead to the development of novel therapeutic strategies, development of biomarkers and further our biologic understanding of the interplay between the innate immune system and the DNA damage response. However, there remains significant work to be performed in understanding the contributions of the innate immune machinery to the DDR and in understanding the crosstalk between its cell-extrinsic and cell-intrinsic regulation of the response to genotoxic stress.

## Data Availability

Not applicable.
